# Quantum mixed phases of a two-dimensional polarized degenerate Fermi gas in an optical cavity

**DOI:** 10.1038/s41598-017-10686-3

**Published:** 2017-09-05

**Authors:** Yanlin Feng, Kuang Zhang, Jingtao Fan, Feng Mei, Gang Chen, Suotang Jia

**Affiliations:** 1 State Key Laboratory of Quantum Optics and Quantum Optics Devices, Institute of Laser spectroscopy, Shanxi University, Taiyuan, 030006 China; 2 Collaborative Innovation Center of Extreme Optics, Shanxi University, Taiyuan, Shanxi 030006 China

## Abstract

The coupling of ultracold fermions to a high-finesse optical cavity can result in novel many-body phenomena, and has attracted significant interests at present. Here we consider a realization of the Fermi-Dicke model with controllable parameters, based on a two-dimensional polarized degenerate Fermi gas coupled to an optical cavity. We analytically investigate the ground-state properties of such system under the mean-field approximation. We find the system can exhibit a rich phase diagram depending on the fermion-photon coupling strength and the atomic resonant frequency. Contrasting to the bosonic counterpart, a first-order quantum phase transition between the superradiant phase and the normal phase featuring two Fermi surfaces can occur for the weak atomic resonant frequency, and there is a unique mixed phase where this normal phase and the superradiant phase coexist. The experimental detection of our results is also discussed.

## Introduction

Cavity quantum electrodynamics (QED) systems, which remarkably illuminate the fundamental interaction between light and matter, have emerged as a novel platform to explore the many-body physics, and promise potential applications in quantum information processing and quantum computing. Recently, the coupling of a Bose-Einstein condensate to a high-finesse optical cavity has been experimentally achieved^1–8^, thus opens a new avenue that combines cavity QED with ultracold atoms. In particular, the bosonic atoms occupying the same quantum state can interact identically with a single-mode quantized field, which can result in a strong collective matter-field interaction. This has led to the remarkable experimental observation^3–5^ of the celebrated second-order quantum phase transition from the normal phase to the superradiant (SR) phase predicted more than 40 years ago^9, 10^. Cavities moreover allow unconventional dynamical optical potentials which can induce a rich variety of strongly correlated many-body phenomena^11^.

While current cavity QED experiments have focused on bosons, there are surging interests in exploring the novel physics arising from the coupling of ultracold fermions to the optical cavity^12–25^. Unlike bosons, fermions obey the Pauli exclusion principle, and two fermions with weak attractive interaction can form Cooper pairs which are responsible for superconductivity^26^. By coupling ultracold fermions to a high-finesse optical cavity, exotic phenomena have been predicted to arise. For example, recent studies on spinless fermions in the cavity-induced dynamical optical potential have revealed the crucial role of the Fermi statistics on the SR phase transition at moderate and high densities^17–19^. It has been shown that the cavity-assisted spin-orbit coupling^27, 28^ can induce a topological SR phase^20^. Moreover, the cavity-induced artificial magnetic field^22^, chiral phases^23^, and non-trivial topological states^24^ have been reported. Interestingly, when fermions in an infinite lattice are gauge coupled to a cavity mode, a SR phase exhibiting a directed particle flow is found to arise above an infinitesimal pumping threshold^25^.

In this report, we consider a two-dimensional (2D) polarized degenerate Fermi gas coupled to a high-finesse optical cavity and realize a Fermi-Dicke model. Our setup relies on two Raman transitions induced by the quantized cavity field and two transverse pumping lasers, and allows for flexible controllability of the Hamiltonian parameters, including the fermion-photon coupling strength and the effective Zeeman field represented by the atomic resonant frequency. Based on this microscopic model, we study the ground state under the mean-field approximation and find several distinct properties compared to its bosonic counterpart^9, 10^. In particular, we predict a first-order quantum phase transition between the SR phase and the normal phase featuring two Fermi surfaces for the weak atomic resonant frequency. As such, the system exhibits a unique mixed phase where the normal and SR phases coexist. Finally, we discuss possible experiment observations of our results.

## Results

### Model and Hamiltonian

**Figure 1:**
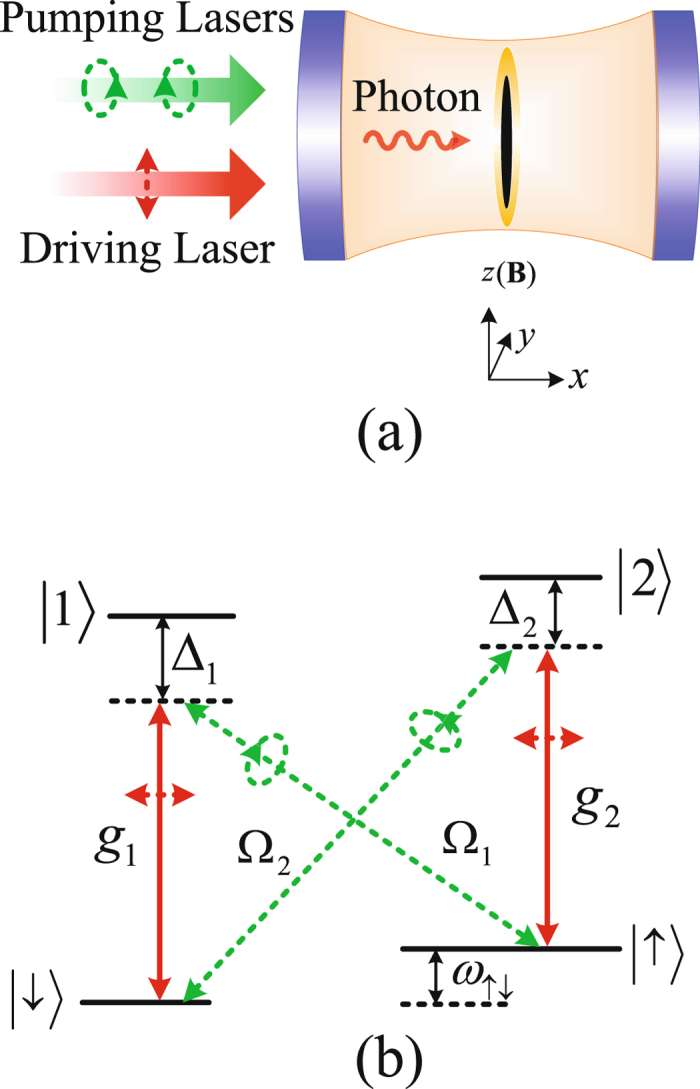
An ensemble of ultracold four-level fermions coupled to a high-finesse optical cavity. (**a**) Schematic of the proposed setup: the ultracold fermions (black online) are confined in a far-of-resonance optical trap (yellow online) in the *yz* plane along with a tightly-radial confinement in the *x* direction. The fermions are coupled to a high-finesse optical cavity, where the cavity mode is driven by a linearly polarized laser (with frequency *ω*_*l*_) propagating along the *x* direction. Two transverse pumping lasers (with frequencies *ω*_*A*_ and *ω*_*B*_), which are left- and right-handed circular polarized in the *yz* plane, propagate along the *x* direction. As a result, two Raman processes are induced. In order to obtain a time-independent Hamiltonian, the condition ${\omega }_{l}=({\omega }_{A}+{\omega }_{B})/2$ is required; see the detailed derivation in the main text. A magnetic field *B* is applied along the positive *z* direction, producing a Zeeman shift between two hyperfine ground states. (**b**) The atomic energy levels and their transitions. Each fermionic atom has two ground states (|↑〉 and |↓〉) and two excited states (|1〉 and |2〉). The |↓〉 ↔|1〉 and |↑〉 ↔|2〉 transitions (red solid lines) are caused by the quantized cavity field with fermion-photon coupling strengths ${g}_{1}$ and ${g}_{2}$. The |↑〉 ↔|1〉 and |↓〉 ↔|2〉 transitions (green dashed lines) are governed by the transverse pumping lasers with Rabi frequencies Ω_1_ and Ω_2_. ${\omega }_{\uparrow \downarrow }={\omega }_{\uparrow }-{\omega }_{\downarrow }$ is the resonant frequency between the ground states |↑〉 and |↓〉 with eigenfrequencies ${\omega }_{\uparrow }$ and ${\omega }_{\downarrow }$. ${{\rm{\Delta }}}_{{\rm{1}}}$ and ${{\rm{\Delta }}}_{2}$ are the detuning from the excited states |1〉 and |2〉.

Motivated by the experiment with bosonic atoms^5^, we instead consider an ensemble of ultracold four-level fermions coupled to a high-finesse optical cavity; see Fig. 1. The fermions are confined in a far-off-resonance optical trap in the *yz* plane [see Fig. 1(a)], and their motion in the *x* direction is frozen, thus effectively realizing a 2D system. The cavity mode is driven by a linearly polarized laser, while the fermions are pumped by two transverse lasers which are left- and right-circular polarized in the *yz* plane, respectively. We consider fermionic atoms with four internal levels, which contain two degenerate ground states (|↑〉 and |↓〉) and two excited states (|1〉 and |2〉) [see Fig. 1(b)]. As clearly illustrated in Fig. 1, in our setup two Raman processes can be induced from the quantized cavity field and the two transverse pumping lasers.

The considered system can be effectively described by the following 2D Hamiltonian

1 $${\hat{H}}_{{\rm{T}}}(t)={\hat{H}}_{{\rm{F}}}+{\hat{H}}_{{\rm{P}}}+{\hat{H}}_{{\rm{D}}}(t)+{\hat{H}}_{{\rm{AR}}}(t)+{\hat{H}}_{{\rm{AP}}},$$

where ${\hat{H}}_{{\rm{F}}}$ is the Hamiltonian for the free four-level fermions, i.e.,

2 $${\hat{H}}_{{\rm{F}}}=\sum _{i\mathrm{=1,2,}\uparrow ,\downarrow }\int {d}^{2}{\bf{r}}{\hat{\psi }}_{i}^{\dagger }({\bf{r}})(\frac{{\hat{{\bf{p}}}}^{2}}{2M}-\mu +{\omega }_{i}){\hat{\psi }}_{i}({\bf{r}})\mathrm{.}$$

Here, ${\hat{\psi }}_{i}^{\dagger }({\bf{r}})$ and ${\hat{\psi }}_{i}({\bf{r}})$ ($i=1,2,\downarrow ,\uparrow$) are the field operators for the fermionic atoms with mass *M*, *μ* is the chemical potential, and *ω*_*i*_ denotes the frequency of the *i*th eigenstate. The quantized cavity field and the driving laser are described by the Hamiltonian

3 $${\hat{H}}_{{\rm{P}}}+{\hat{H}}_{{\rm{D}}}(t)={\omega }_{c}{\hat{a}}^{\dagger }\hat{a}+\varepsilon (\hat{a}{e}^{i{\omega }_{l}t}+{\hat{a}}^{\dagger }{e}^{-i{\omega }_{l}t}),$$

where ${\hat{a}}^{\dagger }$ ($\hat{a}$) creates (annihilates) the quantized cavity field with frequency *ω*_*c*_, and *ε* (*ω*_*l*_) labels the magnitude (frequency) of the driving laser. The Hamiltonian ${\hat{H}}_{{\rm{AR}}}$ represents the interaction between fermions and the transverse pumping lasers, which under the rotating-wave approximation can be written as

4 $${\hat{H}}_{{\rm{AR}}}(t)=\frac{1}{2}\int {d}^{2}{\bf{r}}[{{\rm{\Omega }}}_{1}{\hat{\psi }}_{1}^{\dagger }({\bf{r}}){\hat{\psi }}_{\uparrow }({\bf{r}}){e}^{-i{\omega }_{A}t}+{{\rm{\Omega }}}_{2}{\hat{\psi }}_{2}^{\dagger }({\bf{r}}){\hat{\psi }}_{\downarrow }({\bf{r}}){e}^{-i{\omega }_{B}t}+{\rm{H}}{\rm{.c}}{\rm{.}}],$$

with Ω_1_ and Ω_2_ (*ω*_*A*_ and *ω*_*B*_) being the Rabi frequencies (frequencies) of the two lasers. Finally, Hamiltonian ${\hat{H}}_{{\rm{AP}}}$ describes the interaction between fermions and the quantized cavity fields, i.e.,

5 $${\hat{H}}_{{\rm{AP}}}=\int {d}^{2}{\bf{r}}\{[{g}_{1}{\hat{\psi }}_{1}^{\dagger }({\bf{r}}){\hat{\psi }}_{\downarrow }({\bf{r}})+{g}_{2}{\hat{\psi }}_{2}^{\dagger }({\bf{r}}){\hat{\psi }}_{\uparrow }({\bf{r}})]\hat{a}+{\rm{H}}{\rm{.c}}{\rm{.}}\},$$

with *g*_1_ and *g*_2_ labeling the fermion-photon coupling strengths associated with the two pumping lasers, respectively.

It is more transparent to recast the time-dependent Hamiltonian (1) into a time-independent form. Introducing a unitary transformation $\hat{U}(t)=\exp (i\hat{H}\text{'}t)$, where

6 $$\hat{H}\text{'}={\omega }_{l}{\hat{a}}^{\dagger }\hat{a}+\frac{{\omega }_{B}}{2}[{\hat{\psi }}_{2}^{\dagger }({\bf{r}}){\hat{\psi }}_{2}({\bf{r}})-{\hat{\psi }}_{\downarrow }^{\dagger }({\bf{r}}){\hat{\psi }}_{\downarrow }({\bf{r}})]+\frac{{\omega }_{A}}{2}[{\hat{\psi }}_{1}^{\dagger }({\bf{r}}){\hat{\psi }}_{1}({\bf{r}})-{\hat{\psi }}_{\uparrow }^{\dagger }({\bf{r}}){\hat{\psi }}_{\uparrow }({\bf{r}})]$$

with *ω*_*l*_ = (*ω*_*B*_+*ω*_*A*_)/2, we transform the Hamiltonian as ${\hat{H}}_{1}=\hat{U}(t){\hat{H}}_{{\rm{T}}}(t){\hat{U}}^{\dagger }(t)+i[\partial \hat{U}(t)/\partial t]{\hat{U}}^{\dagger }(t)$. Explicitly, we have

7 $$\begin{array}{c}{\hat{H}}_{1}=\tilde{\omega }{\hat{a}}^{\dagger }\hat{a}+\varepsilon (\hat{a}+{\hat{a}}^{\dagger })+\sum _{i\mathrm{=1,2,}\uparrow ,\downarrow }\int {d}^{2}{\bf{r}}{\hat{\psi }}_{i}^{\dagger }({\bf{r}})(\frac{{\hat{{\bf{p}}}}^{2}}{2M}-\mu ){\hat{\psi }}_{i}({\bf{r}})\\ \quad \quad \,+\int {d}^{2}{\bf{r}}[{{\rm{\Delta }}}_{1}{\hat{\psi }}_{1}^{\dagger }({\bf{r}}){\hat{\psi }}_{1}({\bf{r}})+{{\rm{\Delta }}}_{2}{\hat{\psi }}_{2}^{\dagger }({\bf{r}}){\hat{\psi }}_{2}({\bf{r}})+{\tilde{\omega }}_{\uparrow }{\hat{\psi }}_{\uparrow }^{\dagger }({\bf{r}}){\hat{\psi }}_{\uparrow }({\bf{r}})+{\tilde{\omega }}_{\downarrow }{\hat{\psi }}_{\downarrow }^{\dagger }({\bf{r}}){\hat{\psi }}_{\downarrow }({\bf{r}})]\\ \quad \quad \,+\frac{1}{2}\int {d}^{2}{\bf{r}}[{{\rm{\Omega }}}_{1}{\hat{\psi }}_{1}^{\dagger }({\bf{r}}){\hat{\psi }}_{\uparrow }({\bf{r}})+{{\rm{\Omega }}}_{2}{\hat{\psi }}_{2}^{\dagger }({\bf{r}}){\hat{\psi }}_{\downarrow }({\bf{r}})+{\rm{H}}{\rm{.c}}{\rm{.}}]\\ \quad \quad \,+\int {d}^{2}{\bf{r}}\{[{g}_{1}{\hat{\psi }}_{1}^{\dagger }({\bf{r}}){\hat{\psi }}_{\downarrow }({\bf{r}})+{g}_{2}{\hat{\psi }}_{2}^{\dagger }({\bf{r}}){\hat{\psi }}_{\uparrow }({\bf{r}})]\hat{a}+{\rm{H}}{\rm{.c}}{\rm{.}}\}\mathrm{.}\end{array}$$

Here, $\tilde{\omega }={\omega }_{c}-{\omega }_{l}$ is the effective cavity frequency, ${{\rm{\Delta }}}_{1}={\omega }_{1}-{\omega }_{A}/2$ (${{\rm{\Delta }}}_{2}={\omega }_{2}-{\omega }_{B}\mathrm{/2}$) denotes the detuning from the excited state |1〉 (|2〉), and ${\tilde{\omega }}_{\uparrow }=$ ${\omega }_{\uparrow }+{\omega }_{A}/2$ (${\tilde{\omega }}_{\downarrow }={\omega }_{\downarrow }+{\omega }_{B}/2$) describes the effective frequency associated with the internal state |↑〉 (|↓〉).

To proceed, we recall that in the experiments^3–5^, a weak driving (ε → 0) and large detuning ($|{{\rm{\Delta }}}_{\mathrm{1,2}}|\gg \{{{\rm{\Omega }}}_{\mathrm{1,2}},{g}_{\mathrm{1,2}},\tilde{\omega },{\omega }_{0}\}$) have been considered. Assuming similar scenarios here, this allows us to ignore the term $\varepsilon (\hat{a}+{\hat{a}}^{\dagger })$ in the Hamiltonian (7), as well as adiabatically eliminate both excited states |1〉 and |2〉^29, 30^. This way, we obtain

8 $$\begin{array}{c}\hat{H}=\tilde{\omega }{\hat{a}}^{\dagger }\hat{a}+\sum _{\sigma =\uparrow ,\downarrow }\int {d}^{2}{\bf{r}}{\hat{\psi }}_{\sigma }^{\dagger }({\bf{r}})(\frac{{{\bf{p}}}^{2}}{2M}-\mu ){\hat{\psi }}_{\sigma }({\bf{r}})\\ \quad \quad +\int {d}^{2}{\bf{r}}[{\tilde{\omega }}_{\uparrow }{\hat{\psi }}_{\uparrow }^{\dagger }({\bf{r}}){\hat{\psi }}_{\uparrow }({\bf{r}})+{\tilde{\omega }}_{\downarrow }{\hat{\psi }}_{\downarrow }^{\dagger }({\bf{r}}){\hat{\psi }}_{\downarrow }({\bf{r}})]\\ \quad \quad +\int {d}^{2}{\bf{r}}[\frac{|{g}_{2}{|}^{2}}{{{\rm{\Delta }}}_{2}}{\hat{\psi }}_{\uparrow }^{\dagger }({\bf{r}}){\hat{\psi }}_{\uparrow }({\bf{r}})+\frac{|{g}_{1}{|}^{2}}{{{\rm{\Delta }}}_{1}}{\hat{\psi }}_{\downarrow }^{\dagger }({\bf{r}}){\hat{\psi }}_{\downarrow }({\bf{r}})]{\hat{a}}^{\dagger }\hat{a}\\ \quad \quad +\frac{1}{2}\int {d}^{2}{\bf{r}}\{[\frac{{g}_{2}{{\rm{\Omega }}}_{2}^{\ast }}{{{\rm{\Delta }}}_{2}}{\hat{\psi }}_{\downarrow }^{\dagger }({\bf{r}}){\hat{\psi }}_{\uparrow }({\bf{r}})+\frac{{g}_{1}{{\rm{\Omega }}}_{1}^{\ast }}{{{\rm{\Delta }}}_{1}}{\hat{\psi }}_{\uparrow }^{\dagger }({\bf{r}}){\hat{\psi }}_{\downarrow }({\bf{r}})]\hat{a}+{\rm{H}}{\rm{.c}}{\rm{.}}\}\mathrm{.}\end{array}$$

Further considering

9 $$\frac{|{g}_{1}{|}^{2}}{{{\rm{\Delta }}}_{1}}=\frac{|{g}_{2}{|}^{2}}{{{\rm{\Delta }}}_{2}},\,\frac{{g}_{1}{{\rm{\Omega }}}_{1}^{\ast }}{{{\rm{\Delta }}}_{1}}=\frac{{g}_{2}{{\rm{\Omega }}}_{2}^{\ast }}{{{\rm{\Delta }}}_{2}},$$

the Hamiltonian (8) can be simplified into

10 $$\begin{array}{c}\hat{H}=\omega {\hat{a}}^{\dagger }\hat{a}+\sum _{\sigma =\uparrow ,\downarrow }\int {d}^{2}{\bf{r}}{\hat{\psi }}_{\sigma }^{\dagger }({\bf{r}})(\frac{{\hat{{\bf{p}}}}^{2}}{2M}-\mu ){\hat{\psi }}_{\sigma }({\bf{r}})-{\omega }_{0}\int {d}^{2}{\bf{r}}[{\hat{\psi }}_{\uparrow }^{\dagger }({\bf{r}}){\hat{\psi }}_{\uparrow }({\bf{r}})-{\hat{\psi }}_{\downarrow }^{\dagger }({\bf{r}}){\hat{\psi }}_{\downarrow }({\bf{r}})]\\ \quad \quad +\frac{\eta }{\sqrt{N}}\int {d}^{2}{\bf{r}}[{\hat{\psi }}_{\downarrow }^{\dagger }({\bf{r}}){\hat{\psi }}_{\uparrow }({\bf{r}})+{\hat{\psi }}_{\uparrow }^{\dagger }({\bf{r}}){\hat{\psi }}_{\downarrow }({\bf{r}})](\hat{a}+{\hat{a}}^{\dagger })\mathrm{.}\end{array}$$

Here, the factor $1/\sqrt{N}$, with *N* being the total atom number, is introduced so as to ensure a finite free energy per fermion in the thermodynamic limit^9, 10^. Furthermore, *ω*_0_ = $({\tilde{\omega }}_{\downarrow }-{\tilde{\omega }}_{\uparrow })\mathrm{/2}$ is the effective resonant frequency between the ground states |↑〉 and |↓〉, which in Eq. (10) acts as an effective Zeeman field. Without loss of generality, below we shall take ${\omega }_{0} > 0$. In addition, the parameter $\eta =\sqrt{N}{g}_{1}{{\rm{\Omega }}}_{1}^{\ast }/(2{{\rm{\Delta }}}_{1})$ $=\sqrt{N}{g}_{2}{{\rm{\Omega }}}_{2}^{\ast }/(2{{\rm{\Delta }}}_{2})$ is the effective fermion-photon coupling strength, and $\omega =N\zeta +\tilde{\omega }$ labels the atom-number dependent cavity frequency with $\zeta =|{g}_{1}{|}^{2}{/{\rm{\Delta }}}_{1}=|{g}_{2}{|}^{2}/{{\rm{\Delta }}}_{2}$. We remark that all the parameter choice here are motivated by the experimental considerations^3–5^.

The Hamiltonian (10) represents the paradigmatic Fermi-Dicke model^31^ describing the fermion-photon interaction. We emphasize that our setup allows flexible controllability of all Hamiltonian parameters. For example, both *ω*_0_ and *ω* can be tuned by modifying the frequencies of the driving laser and the transverse pumping lasers, while *η* can be controlled via the Rabi frequencies of the transverse pumping lasers.

### Ground-state properties

Our goal is to investigate the ground state of the Hamiltonian (10). To this end, it is more convenient to transform to the momentum space representation. Writing

11 $${\hat{\psi }}_{\sigma }({\bf{r}})=\frac{1}{\sqrt{S}}\sum _{{\bf{k}}{\boldsymbol{,}}\sigma =\uparrow ,\downarrow }{\hat{C}}_{{\bf{k}},\sigma }{e}^{i{\bf{k}}\cdot {\bf{r}}},$$

where ${\hat{C}}_{{\bf{k}},\sigma }$ annihilates a fermion in the internal state *σ* with momentum **k** and *S* is the system size (hereafter *S* = 1 is set for convenience), we obtain

12 $$\begin{array}{c}\hat{H}=\omega {\hat{a}}^{\dagger }\hat{a}+\sum _{{\bf{k}}}{\xi }_{{\bf{k}}}{\hat{C}}_{{\bf{k}},\sigma }^{\dagger }{\hat{C}}_{{\bf{k}},\sigma }+{\omega }_{0}\sum _{{\bf{k}}}({\hat{C}}_{{\bf{k}},\uparrow }^{\dagger }{\hat{C}}_{{\bf{k}},\uparrow }-{\hat{C}}_{{\bf{k}},\downarrow }^{\dagger }{\hat{C}}_{{\bf{k}},\downarrow })\\ \quad \quad +\frac{\eta }{\sqrt{n}}\sum _{{\bf{k}}}({\hat{C}}_{{\bf{k}},\uparrow }^{\dagger }{\hat{C}}_{{\bf{k}},\downarrow }+{\hat{C}}_{{\bf{k}},\downarrow }^{\dagger }{\hat{C}}_{{\bf{k}},\uparrow })(\hat{a}+{\hat{a}}^{\dagger })\mathrm{.}\end{array}$$

Here, ${\xi }_{{\bf{k}}}={\varepsilon }_{{\bf{k}}}-\mu$, ${\varepsilon }_{{\bf{k}}}={{\bf{k}}}^{2}/2M$ is the kinetic energy, and $n={K}_{F}^{2}/(2\pi )={E}_{F}M/\pi$ is the 2D density of fermions with ${E}_{F}={K}_{F}^{2}/(2M)$ being the Fermi energy and *K*_*F*_ being the Fermi momentum.

In solving the ground state of the Hamiltonian (12), we will rely on the mean-field approximation, i.e., by replacing $\hat{a}$ with its steady-state value. Specifically, we write down the Heisenberg-Langevin equation for the cavity field operator $\hat{a}$ ^32, 33^, taking into account of the cavity decay with rate *κ*, i.e.,

13 $$i\frac{\partial \hat{a}}{\partial t}=[\hat{a},\hat{H}]-i\kappa \hat{a}+{\hat{\gamma }}_{{\rm{in}}}(t)\mathrm{.}$$

For the quantum noise operator ${\hat{\gamma }}_{{\rm{in}}}(t)$, we have $\langle {\hat{\gamma }}_{{\rm{in}}}^{\dagger }(t){\hat{\gamma }}_{{\rm{in}}}(t\text{'})\rangle =2\kappa \delta (t-t\text{'})$ and $\langle {\hat{\gamma }}_{{\rm{in}}}(t){\hat{\gamma }}_{{\rm{in}}}(t\text{'})\rangle =0$. Since quantum noise usually occurs on a much shorter time scale than 1/*κ* ^34^, its average effect can be generically ignored on the time scale relevant for the steady state. When 1/*κ* is much shorter than the time scales of system dynamics, a steady-state solution^3, 35^ to Eq. (13) can be found, i.e.,

14 $$\alpha =\langle \hat{a}\rangle =\frac{\eta \sum _{k}\langle {\hat{C}}_{{\bf{k}},\uparrow }^{\dagger }{\hat{C}}_{{\bf{k}},\downarrow }+{\hat{C}}_{{\bf{k}},\downarrow }^{\dagger }{\hat{C}}_{{\bf{k}},\uparrow }\rangle }{\sqrt{n}(-\omega +i\kappa )}\mathrm{.}$$

Motivated by the experiments^3, 4^ which shows that the mean-photon number $\langle {\hat{a}}^{\dagger }\hat{a}\rangle ={|\alpha |}^{2}$ determines the SR properties, we henceforth refer to it as the SR order parameter.

By approximating $\hat{a}\approx \langle \hat{a}\rangle$ in Eq. (12) using Eq. (14), we obtain a quadratic Hamiltonian

15 $$\hat{H}=\sum _{{\bf{k}}}{\hat{{\rm{\Psi }}}}_{{\bf{k}}}^{\dagger }{M}_{{\bf{k}}}{\hat{{\rm{\Psi }}}}_{{\bf{k}}}+\omega {|\alpha |}^{2}\mathrm{.}$$

Here, ${\hat{{\rm{\Psi }}}}_{{\bf{k}}}={({\hat{C}}_{{\bf{k}},\uparrow },{\hat{C}}_{{\bf{k}},\downarrow })}^{T}$ is the standard Nambu spinor, and *M*_**k**_ is the photon-number dependent BdG matrix, i.e.,

16 $${M}_{{\bf{k}}}=(\begin{array}{cc}{\xi }_{{\bf{k}}}-{\omega }_{0} & \bar{\eta }\\ \bar{\eta } & {\xi }_{{\bf{k}}}+{\omega }_{0}\end{array}),$$

with $\bar{\eta }=\eta (\alpha +{\alpha }^{\ast })/\sqrt{n}$. Now, the Hamiltonian (15) can be easily diagonalized as

17 $$\hat{H}=\sum _{{\bf{k}}}({E}_{{\bf{k}},+}{\hat{\alpha }}_{{\bf{k}},+}^{\dagger }{\hat{\alpha }}_{{\bf{k}},+}+{E}_{{\bf{k}},-}{\hat{\alpha }}_{{\bf{k}},-}^{\dagger }{\hat{\alpha }}_{{\bf{k}},-})+\omega {|\alpha |}^{2},$$

where ${\hat{\alpha }}_{{\bf{k}},\pm }$ describe the fermionic Bogoliubov quasiparticles, whose energy is given by

18 $${E}_{{\bf{k}},\pm }={\xi }_{{\bf{k}}}\pm \sqrt{{\bar{\eta }}^{2}+{\omega }_{0}^{2}},$$

with ${\bar{\eta }}^{2}=4{\omega }^{2}{\eta }^{2}{|\alpha |}^{2}/[n({\omega }^{2}+{\kappa }^{2})]$. Note that ${E}_{{\bf{k}},\pm }$ can be either positive or negative depending on parameter choices. Regrouping terms with positive and negative energies using the Heaviside step function ${\rm{\Theta }}(x)$, we recast Eq. (17) as

19 $$\hat{H}=\sum _{{\bf{k}}{\boldsymbol{,}}\pm }{E}_{{\bf{k}},\pm }{\rm{\Theta }}({E}_{{\bf{k}},\pm }){\hat{\alpha }}_{{\bf{k}},\pm }^{\dagger }{\hat{\alpha }}_{{\bf{k}},\pm }-\sum _{{\bf{k}}{\boldsymbol{,}}\pm }{E}_{{\bf{k}},\pm }{\rm{\Theta }}(-{E}_{{\bf{k}},\pm }){\hat{\alpha }}_{{\bf{k}},\pm }{\hat{\alpha }}_{{\bf{k}},\pm }^{\dagger }+{E}_{{\rm{G}}},$$

where *E*_*G*_ is the ground-state energy expressed by

20 $${E}_{{\rm{G}}}=\sum _{{\bf{k}}}{E}_{{\bf{k}},+}{\rm{\Theta }}(-{E}_{{\bf{k}},+})+{E}_{{\bf{k}},-}{\rm{\Theta }}(-{E}_{{\bf{k}},-})+\omega {|\alpha |}^{2}\mathrm{.}$$

Despite the formal expression of the ground-state energy, Eq. (20) contains two variables, i.e., *μ* and |*α*|, which are to be determined from the particle number equation $\partial {E}_{{\rm{G}}}/\partial \mu =-n$ and the SR equation $\partial {E}_{{\rm{G}}}/\partial (|\alpha |)=0$. Explicitly, we have

21 $$\sum _{{\bf{k}}}(-1)[f(-{E}_{{\bf{k}},+})+f(-{E}_{{\bf{k}},-})]=n,$$

22 $$|\alpha |\{\sum _{{\bf{k}}}-2\omega {\eta }^{2}[f(-{E}_{{\bf{k}},-})-f(-{E}_{{\bf{k}},+})]+n\bar{\chi }({\omega }^{2}+{\kappa }^{2})\}=\mathrm{0,}$$

where $f(-{E}_{{\bf{k}},\pm })={\rm{\Theta }}(-{E}_{{\bf{k}},\pm })-{E}_{{\bf{k}},\pm }\delta (-{E}_{{\bf{k}},\pm })$ and $\bar{\chi }=\sqrt{{\bar{\eta }}^{2}+{\omega }_{0}^{2}}$. In deriving Eqs (21) and (22), we have used the identity ${\rm{\Theta }}\text{'}(x)=\delta (x)$ with δ(*x*) being the delta function.

Equations (20–22) allow complete specification of the ground state: The ground-state energy can be obtained in a *self-consistent* manner by solving Eqs (21) and (22) for a fixed atom density *n*. It is important to bear in mind that solutions of above equations must be supplemented with a careful stability analysis, which we describe below.

### Phase diagram

We now detail our analysis on the ground-state properties. We shall be interested in four quantities: the ground-state energy per fermion ${\bar{E}}_{{\rm{G}}}={E}_{{\rm{G}}}/n$, the chemical potential *μ*, the scaled mean-photon number ${|\bar{\alpha }|}^{2}$, and the scaled polarization^36^ measuring the response to the effective Zeeman field defined by

23 $$\bar{m}=\frac{{n}_{\uparrow }-{n}_{\downarrow }}{n}=-\frac{\partial {\bar{E}}_{{\rm{G}}}}{\partial {\omega }_{0}}\mathrm{.}$$

As we shall see, their ground-state values depend crucially on the strength of the effective Zeeman field *ω*_0_ and the effective fermion-photon coupling strength *η*, in particular, new features compared to their boson counterpart are found in the regime ${\omega }_{0} < {E}_{F}$. Note that all energies will be measured in units of *E*_*F*_ hereupon.

In order to gain some intuition, let us first consider the simplest case *η* = 0 corresponding to a free Fermi gas. Transforming Eq. (20) into an integral over momenta as usual, we calculate the ground-state energy per fermion as

24 $${\bar{E}}_{{\rm{G}}}=-\frac{1}{4{E}_{F}}[{(\mu +{\omega }_{0})}^{2}{\rm{\Theta }}(\mu +{\omega }_{0})+{(\mu -{\omega }_{0})}^{2}{\rm{\Theta }}(\mu -{\omega }_{0})]\mathrm{.}$$

Because $\mu +{\omega }_{0} < 0$ will entail a unphysical result ${\bar{E}}_{{\rm{G}}}=0$ which excludes existence of real fermions^37, 38^, we shall limit our subsequent discussions in the regime $\mu +{\omega }_{0} > 0$. There, the Fermi gas can exhibit different ground states depending on whether ${\omega }_{0} > \mu$ or ${\omega }_{0} < \mu$.

When ${\omega }_{0} > \mu$, ${\rm{\Theta }}(\mu -{\omega }_{0})=0$ and Eq. (24) becomes

25 $${\bar{E}}_{{\rm{G}}}=-\frac{{({\omega }_{0}+\mu )}^{2}}{4{E}_{F}}\mathrm{.}$$

Based on Eqs (21), (23) and (25), we obtain

26 $${\bar{E}}_{{\rm{G}}}=-{E}_{F},\,\mu =2{E}_{F}-{\omega }_{0},\,\bar{m}=1.$$

Thus the Fermi gas is in a normal phase characterized by a full polarization and one Fermi surface with ${\mu }_{\uparrow }=2{E}_{F}$, and we shall refer to it as the N-I phase.

In contrast, when ${\omega }_{0} < \mu$, ${\rm{\Theta }}(\mu -{\omega }_{0})=1$ and Eq. (24) becomes

27 $${\bar{E}}_{{\rm{G}}}=-\frac{{\omega }_{0}^{2}+{\mu }^{2}}{4{E}_{F}}\mathrm{.}$$

Based on Eqs (21), (23) and (25), we have

28 $${\bar{E}}_{{\rm{G}}}=-\frac{({E}_{F}^{2}+{\omega }_{0}^{2})}{2{E}_{F}},\,\mu ={E}_{F},\,\bar{m}=\frac{{\omega }_{0}}{{E}_{F}}\mathrm{.}$$

Hence the Fermi gas is only partially polarized and exhibits two Fermi surfaces, i.e., ${\mu }_{\uparrow }={E}_{F}+{\omega }_{0}$ and ${\mu }_{\downarrow }={E}_{F}-{\omega }_{0}$. We shall call it as the N-II phase. It is clear from Eqs (26) and (28) that, by tuning the parameter *ω*_0_ across the critical point ${\omega }_{0}^{c}={E}_{F}$ ^37^, the Fermi gas undergoes a first-order transition between the N-I to N-II phases.

Now, suppose there exists a weak effective coupling between the photon and fermions, i.e., *η* is small. Still, the noninteracting terms in the Hamiltonian (12) dominate the system dynamics, such that |*α*|^2^ = 0 is expected to persist for the ground state and the Fermi gas remains in the N-I or N-II phase (depending on values of *ω*_0_).

However, the ground-state properties of the Fermi gas can change drastically from both normal phases for a strong photon-fermion coupling when *η* is increased above a threshold. As we show now, when the interaction part of the Hamiltonian (12) dominates, the system can acquire a macroscopic collective excitation with |*α*|^2^ ≠ 0, i.e., a quantum phase transition into the SR phase occurs when *η* is above some critical values. Integrating Eq. (20) over the momenta, we obtain

29 $${\bar{E}}_{{\rm{G}}}=-\frac{1}{4{E}_{F}}[{\mu }_{+}^{2}+{\mu }_{-}^{2}{\rm{\Theta }}({\mu }_{-})]+\omega {|\bar{\alpha }|}^{2},$$

with ${\mu }_{\pm }=\mu \pm \bar{\chi }$ and ${|\bar{\alpha }|}^{2}={|\alpha |}^{2}/n$ being the scaled mean-photon number. We next analyze Eq. (29) when (i) ${\mu }_{-} < 0$ and (ii) ${\mu }_{-}\ge 0$.

Consider first the case ${\mu }_{-} < 0$, where Eq. (29) becomes

30 $${\bar{E}}_{{\rm{G}}}=-\frac{1}{4{E}_{F}}{\mu }_{+}^{2}+\omega {|\bar{\alpha }|}^{2},$$

and Eqs (21–23) take the following form

31 $$\mu +\sqrt{{\bar{\eta }}^{2}+{\omega }_{0}^{2}}=2{E}_{F},$$

32 $$|\bar{\alpha }|[2\omega -\frac{2{\omega }^{2}{\eta }^{2}(\mu +\sqrt{{\bar{\eta }}^{2}+{\omega }_{0}^{2}})}{{E}_{F}({\omega }^{2}+{\kappa }^{2})\sqrt{{\bar{\eta }}^{2}+{\omega }_{0}^{2}}}]=\mathrm{0,}$$

33 $$\frac{{\omega }_{0}(\mu +\sqrt{{\bar{\eta }}^{2}+{\omega }_{0}^{2}})}{2{E}_{F}\sqrt{{\bar{\eta }}^{2}+{\omega }_{0}^{2}}}=\bar{m}\mathrm{.}$$

We can obtain two set of solutions, namely,

34 $$\mu =2{E}_{F}-{\omega }_{0},\,|\bar{\alpha }|=0,\,\bar{m}=\mathrm{1,}$$

or

35 $$\mu =2({E}_{F}-\frac{\omega {\eta }^{2}}{{\omega }^{2}+{\kappa }^{2}}),\,|\bar{\alpha }|=\sqrt{\frac{{\eta }^{2}}{{\omega }^{2}+{\kappa }^{2}}-\frac{{\omega }_{0}^{2}({\omega }^{2}+{\kappa }^{2})}{4{\omega }^{2}{\eta }^{2}}},\,\bar{m}=\frac{{\omega }_{0}({\omega }^{2}+{\kappa }^{2})}{2\omega {\eta }^{2}}\mathrm{.}$$

Obviously, while solutions (34) are associated with the normal phase, solutions (35) exhibit SR property. Furthermore, as mentioned earlier, the ground-state solutions must satisfy the stability condition as defined by ${\partial }^{2}{\bar{E}}_{{\rm{G}}}/\partial {(|\bar{\alpha }|)}^{2} > 0$. Consequently, we find that the stability condition for the normal solution [see Eq. (34)] is $\eta  < {\eta }_{c}^{\mathrm{(1)}}$ with

36 $${\eta }_{c}^{\mathrm{(1)}}=\sqrt{\frac{{\omega }_{0}({\omega }^{2}+{\kappa }^{2})}{2\omega }}\mathrm{.}$$

Instead, the SR solution [see Eq. (35)] is only stable when $\eta  > {\eta }_{c}^{\mathrm{(1)}}$. Notice that since ${\mu }_{-}=\mu -\bar{\chi } < 0$, this additionally requires $\eta  > {\eta }_{c}^{\mathrm{(2)}}$ with

37 $${\eta }_{c}^{\mathrm{(2)}}=\sqrt{\frac{{E}_{F}({\omega }^{2}+{\kappa }^{2})}{2\omega }}\mathrm{.}$$

**Figure 2:**
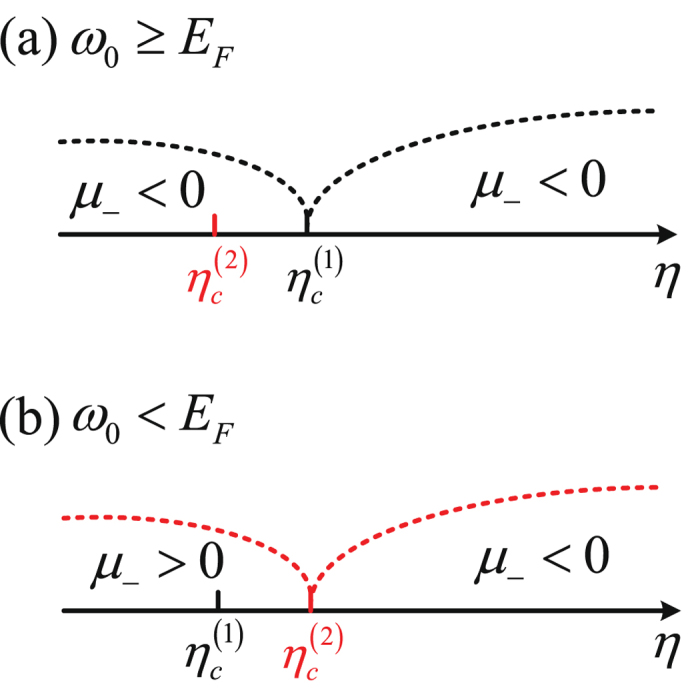
Comparison of the critical points ${\eta }_{c}^{(1)}$ and ${\eta }_{c}^{(2)}$ for (a) ${\omega }_{0}\ge {E}_{F}$ and (b) ${\omega }_{0} < {E}_{F}$. When ${\omega }_{0}\ge {E}_{F}$, ${\eta }_{c}^{\mathrm{(1)}} > {\eta }_{c}^{\mathrm{(2)}}$, and ${\mu }_{-} < 0$ for both $0 < \eta  < {\eta }_{c}^{\mathrm{(1)}}$ and $\eta  > {\eta }_{c}^{\mathrm{(1)}}$. When ${\omega }_{0} < {E}_{F}$, ${\eta }_{c}^{\mathrm{(1)}} < {\eta }_{c}^{\mathrm{(2)}}$, and ${\mu }_{-} < 0$ for $\eta  > {\eta }_{c}^{\mathrm{(2)}}$ and ${\mu }_{-} > 0$ for $0 < \eta  < {\eta }_{c}^{\mathrm{(2)}}$.

Following from above analysis, we find that for ${\omega }_{0}\ge {E}_{F}$ [c.f. Fig. 2(a)] and thereby ${\eta }_{c}^{\mathrm{(1)}} > {\eta }_{c}^{\mathrm{(2)}}$, the ground state of the Fermi gas is in the N-I phase for $\mathrm{0 < }\eta  < {\eta }_{c}^{\mathrm{(1)}}$, but transits into the SR phase for $\eta  > {\eta }_{c}^{\mathrm{(1)}}$; see summarized results below

38 $${\bar{E}}_{{\rm{G}}}=\{\begin{array}{cc}-{E}_{F} & 0 < \eta  < {\eta }_{c}^{\mathrm{(1)}}\\ -{E}_{F}+\frac{\omega {\eta }^{2}}{{\omega }^{2}+{\kappa }^{2}}-\frac{{\omega }_{0}^{2}({\omega }^{2}+{\kappa }^{2})}{4\omega {\eta }^{2}} & \eta  > {\eta }_{c}^{\mathrm{(1)}}\end{array},$$

with corresponding relevant quantities given by

39 $$\mu =\{\begin{array}{ll}2{E}_{F}-{\omega }_{0} & 0 < \eta  < {\eta }_{c}^{\mathrm{(1)}}\\ 2({E}_{F}-\frac{\omega {\eta }^{2}}{{\omega }^{2}+{\kappa }^{2}}) & \eta  > {\eta }_{c}^{\mathrm{(1)}}\end{array},$$

40 $$|\bar{\alpha }|=\{\begin{array}{ll}0 & 0 < \eta  < {\eta }_{c}^{\mathrm{(1)}}\\ \sqrt{\frac{{\eta }^{2}}{{\omega }^{2}+{\kappa }^{2}}-\frac{{\omega }_{0}^{2}({\omega }^{2}+{\kappa }^{2})}{4{\omega }^{2}{\eta }^{2}}} & \eta  > {\eta }_{c}^{\mathrm{(1)}}\end{array},$$

41 $$\bar{m}=\{\begin{array}{ll}1 & 0 < \eta  < {\eta }_{c}^{\mathrm{(1)}}\\ \frac{{\omega }_{0}({\omega }^{2}+{\kappa }^{2})}{2\omega {\eta }^{2}} & \eta  > {\eta }_{c}^{\mathrm{(1)}}\end{array}.$$

**Figure 3:**
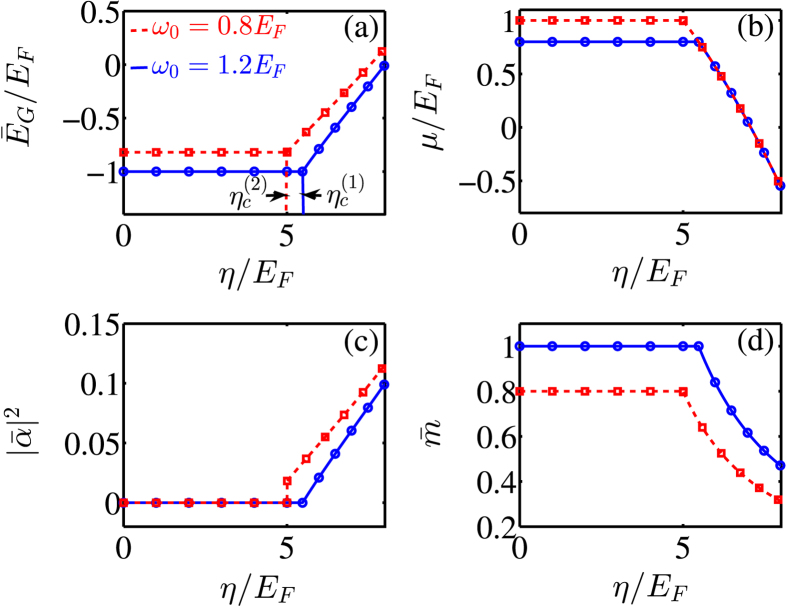
(**a**) The scaled ground-state energy ${\bar{E}}_{{\rm{G}}}/{E}_{F}$, (**b**) the chemical potential $\mu /{E}_{F}$, (**c**) the scaled mean-photon number ${|\bar{\alpha }|}^{2}$, and (**d**) the scaled polarization $\bar{m}$ as functions of the effective atom-photon coupling strength $\eta /{E}_{F}$. Analytical results are depicted by blue-solid and red-dashed lines, while the open symbols represent numerical simulations. For the atom-number dependent cavity frequency, we choose $\omega =10{E}_{F}$, and we take the cavity decay rate as $\kappa =20{E}_{F}$. For ${\omega }_{0}=1.2{E}_{F}$, we take ${\eta }_{c}^{\mathrm{(1)}}=5.48{E}_{F}$, and when $\,{\omega }_{0}=0.8{E}_{F}$, we take ${\eta }_{c}^{\mathrm{(2)}}=$ $5{E}_{F}$.

The ground-state properties described by Eqs (38)–(41) exhibit two features. First, the quantum phase transition between the N-I and SR phases is second order in nature: while the first-order derivative of ${\bar{E}}_{{\rm{G}}}$ is continuous with respect to *η*, its second order derivative develops discontinuity at the critical point ${\eta }_{c}^{\mathrm{(1)}}$, as in the case with ultracold Bose atoms^9, 10^. Second, in contrast to the N-I phase, the SR phase features macroscopic collective excitations for both fermions and photons, i.e., we have both $|\bar{\alpha }|\ne 0$ and $\bar{m}\ne 0$. Also note that while $\bar{m}\sim {\eta }^{2}$, we see $\mu \sim {\eta }^{-2}$. These analytical results are confirmed by our numerical calculations as illustrated by Fig. 3, where we plot ${\bar{E}}_{{\rm{G}}}$, *μ*, ${|\bar{\alpha }|}^{2}$, and $\bar{m}$ as functions of *η*, respectively.

Next, turning to the case ${\mu }_{-}=\mu -\bar{\chi }\ge 0$, we find from Eq. (29) that

42 $${\bar{E}}_{{\rm{G}}}=-\frac{1}{4{E}_{F}}({\mu }_{+}^{2}+{\mu }_{-}^{2})+\omega {|\bar{\alpha }|}^{2}\mathrm{.}$$

Imposing the stability condition ${\partial }^{2}{\bar{E}}_{{\rm{G}}}/\partial {(|\bar{\alpha }|)}^{2} > 0$, and keeping in mind ${\mu }_{-}\ge 0$, we find that in the regimes ${\omega }_{0} < {E}_{F}$ and $0 < \eta  < {\eta }_{c}^{\mathrm{(2)}}$, the relevant ground-state solutions are

43 $$\mu ={E}_{F},\,|\bar{\alpha }|=0,\,\bar{m}=\frac{{\omega }_{0}}{{E}_{F}}\mathrm{.}$$

Note that for ${\omega }_{0} < {E}_{F}$ and $\eta  > {\eta }_{c}^{\mathrm{(2)}}$, we would have ${\mu }_{-} < 0$ [c.f. Fig. 2(b)], where the ground-state solutions are of the form of Eq. (35). Thus in contrast to the case ${\omega }_{0}\ge {E}_{F}$, the ground state of the Fermi gas with ${\omega }_{0} < {E}_{F}$ exhibits the N-II phase for $0 < \eta  < {\eta }_{c}^{(2)}$ and the SR phase for $\eta  > {\eta }_{c}^{\mathrm{(2)}}$. We summarize results below

44 $${\bar{E}}_{{\rm{G}}}=\{\begin{array}{ll}-\frac{{E}_{F}}{2}-\frac{{\omega }_{0}^{2}}{2{E}_{F}} & 0 < \eta  < {\eta }_{c}^{\mathrm{(2)}}\\ -{E}_{F}+\frac{\omega {\eta }^{2}}{{\omega }^{2}+{\kappa }^{2}}-\frac{{\omega }_{0}^{2}({\omega }^{2}+{\kappa }^{2})}{4\omega {\eta }^{2}} & \eta  > {\eta }_{c}^{\mathrm{(2)}}\end{array}.$$

We see that a quantum phase transition from the N-II phase to the SR phase occurs at the critical point ${\eta }_{c}^{\mathrm{(2)}}$, which is first order in nature and is accompanied by a sudden change in ${|\bar{\alpha }|}^{2}$ [see also red-dashed curves in Fig. 3(c)].

Interestingly, at the critical regime $\eta ={\eta }_{c}^{(2)}$, the scaled ground-state energies of the N-II and SR phases become equal, meaning both phases coexist. We shall therefore call it *the N-II-SR mixed phase*. In order to characterize this mixed phase, let *x*_0_ stand for the fraction of the N-II part in the mixed phase, which can take arbitrary value in the regime $0\le {x}_{0}\le 1$. We find (see Methods Section for detailed derivation)

45 $${\bar{E}}_{{\rm{G}}}=\mu -\frac{{x}_{0}({\omega }_{0}^{2}+{E}_{F}^{2})}{2{E}_{F}}-\frac{(1-{x}_{0})}{2}({E}_{F}+\frac{{\omega }_{0}^{2}}{{E}_{F}}),$$

46 $$\mu ={E}_{F},$$

47 $$|\bar{\alpha }|=\sqrt{\frac{{E}_{F}^{2}-{\omega }_{0}^{2}}{2\omega {E}_{F}}},$$

48 $$\bar{m}=\frac{{x}_{0}{\omega }_{0}}{{E}_{F}}+(1-{x}_{0})\frac{{\omega }_{0}({\omega }^{2}+{\kappa }^{2})}{2\omega {\eta }^{2}}\mathrm{.}$$

Since both the SR and N-II phases exhibit $\bar{m}\ne 0$ [see Eqs (35) and (28)], the former due to the the macroscopic collective excitation of fermions while the later induced by the effective Zeeman field, it is naturally expected that both mechanisms contribute to the nonzero polarization of the N-II-SR mixed phase [see Eq. (48)]. In addition, we find two first-order quantum phase transitions, from the N-II-SR phase to the N-II phase and to the SR phase, respectively. This is different from the boson counterpart, as well as the previously discussed N-I phase, where the corresponding transitions are second order^9, 10^.

**Figure 4:**
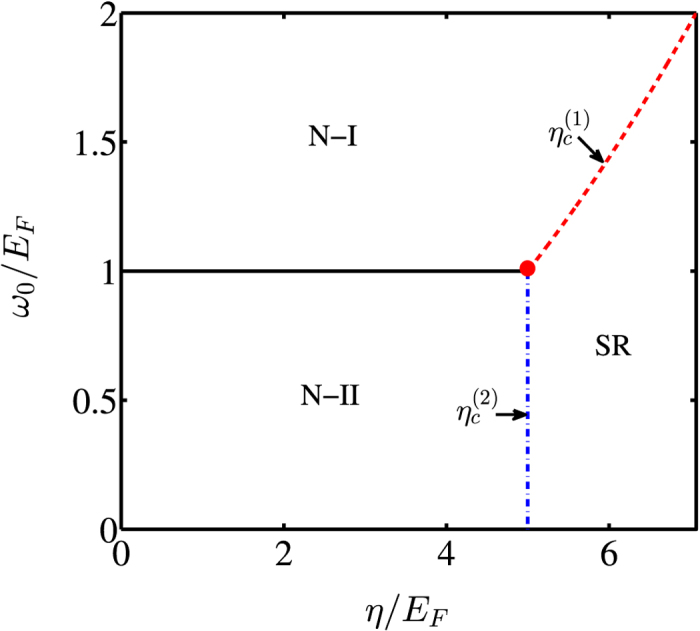
Phase diagram as a function of the effective resonant frequency ${\omega }_{0}/{E}_{F}$ and the effective fermion-photon coupling strength $\eta /{E}_{F}$. The atom-number dependent cavity frequency ω and the cavity decay rate *κ* are the same as those in Fig. 3. When the effective resonant frequency is chosen as ${\omega }_{0}={E}_{F}$, ${\eta }_{c}^{(1)}={\eta }_{c}^{(2)}$.

Collecting all above results, we plot in Fig. 4 the phase diagram in the entire parameter regimes of ${\omega }_{0}/{E}_{F}$ and $\eta /{E}_{F}$. As predicted previously, while the quantum phase transition from the N-I phase to the SR phase is second order, a first-order transition occurs between the N-II and SR phases due to coexistence of both phases at the critical line. In addition, the phase diagram exhibits a tri-critical point (see the red dotted curves), where the quantum phase transition changes its character from the first to the second order.

### Parameter estimation and possible experimental observation

We now provide an estimation of the relevant parameters taking ^40^K atom as the example. For the fermionic ^40^K atoms with the Fermi energy ${E}_{F}\sim 0.73$ MHz, the ground states with ^2^S_1/2_ are represented by |↑〉 = |*F* = 9/2, *m*_*F*_ = 9/2〉 and |↓〉 = |*F* = 9/2, *m*_*F*_ = 7/2〉. For the excited states with ^2^P_1/2_, we choose |1〉 = |*F* = 9/2, *m*_*F*_ = 7/2〉 and |2〉 = |*F* = 9/2, *m*_*F*_ = 9/2〉, with *F* and m_*F*_ labeling the total angular momentum and magnetic quantum numbers, respectively.

Considering the optical properties of the ^40^K D1-line, we take the cavity length as 178 *μ*m and the wavelengths of the transverse pumping lasers as 770 nm. This ensures that both *g*_1_ and *g*_2_ characterizing the fermion-photon coupling strength are at the order of MHz, such that the rotating-wave approximation leading to Eqs (4) and (5) is valid. For a cavity with a waist radius 27 *μ*m and a finesse ~10^5^, we estimate its decay rate *κ* is at the order of MHz. For the effective fermion-photon coupling strength $\eta =\sqrt{N}{g}_{1}{{\rm{\Omega }}}_{1}^{\ast }/(2{{\rm{\Delta }}}_{1})$ $=\sqrt{N}{g}_{2}{{\rm{\Omega }}}_{2}^{\ast }/(2{{\rm{\Delta }}}_{2})$, thanks to the prefactor $\sqrt{N}$, a magnitude of MHz can be achieved simply by modifications of the Rabi frequencies of the transverse pumping lasers, despite the large detuning required by the adiabatic approximation leading to Eq. (8). Finally, the effective resonant frequency *ω*_0_ = $({\tilde{\omega }}_{\downarrow }-{\tilde{\omega }}_{\uparrow })/2$ and the atom-number dependent cavity frequency $\omega =N\zeta +\tilde{\omega }$ can be easily controlled by tuning the frequencies of the driving and transverse pumping lasers. In the experiments^3^, both *ω*_0_ and *ω* can be tuned from − GHz to GHz and even beyond.

We conclude this section by briefly discussing how to probe the predicted quantum phases and phase diagrams. As has been elaborated, the physics of the ground-state phases is determined by the scaled mean-photon number ${|\bar{\alpha }|}^{2}$ and the scaled polarization $\bar{m}$. Building on the development of the state of the art experimental techniques, $\bar{m}$ can be measured by observing the different density distributions between the two-component Fermi gas^39, 40^, while *α* can be detected using the calibrated single-photon counting modules which allows for the *in situ* monitor of the intra-cavity light intensity^3^. We thus expect that our results to be feasible within the experimental capabilities.

## Discussion

In realistic experiments, there typically exists a shallow harmonic external confining potential, which can be modeled by $V(r)={\omega }_{\perp }^{2}{r}^{2}/2$, where ${\omega }_{\perp }$ is the harmonic trap frequency and *r* is the harmonic trap radius. In order to estimate its effect, we first use the local density approximation^41^ to obtain an effective chemical potential $\mu (r)={\mu }_{0}-V(r)$, where *μ*_0_ is the chemical potential at the center of the harmonic trap and *μ*(*r*) determines the total density $n={n}_{\uparrow }+{n}_{\downarrow }$. Then, the density distribution *n*(*r*) and the magnetization distribution *m*(*r*) can be solved from^42^

49 $$N=2\pi \int rdrn(r),\,\bar{m}=\frac{2\pi }{N}\int rdrm(r)\mathrm{.}$$

**Figure 5:**
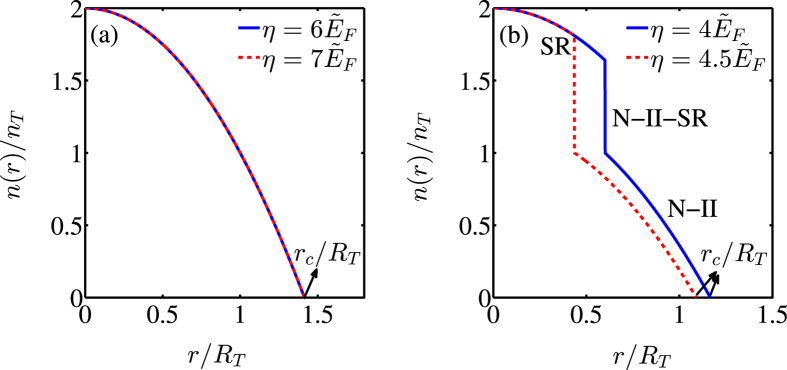
The density distributions $n(r)/{n}_{0}$ for (**a**) ${\omega }_{0}\ge {\mathop{E}\limits^{ \sim }}_{F}$ and (**b**) ${\omega }_{0} < {\mathop{E}\limits^{ \sim }}_{F}$ along the radial direction $r/{R}_{T}$. The atom-number dependent cavity frequency *ω* and the cavity decay rate *κ* are the same as those in Fig. 3. The scaled polarization is taken as $\bar{m}=0.5$.

In Fig. 5, we plot the density distributions *n*(*r*)/*n*_0_ in the radial direction for (a) ${\omega }_{0}\ge {\tilde{E}}_{F}$ and (b) ${\omega }_{0} < {\tilde{E}}_{F}$, respectively, where ${n}_{0}=M{\tilde{E}}_{F}/(2\pi )$, ${R}_{T}=\sqrt{2{\tilde{E}}_{F}}/{\omega }_{\perp }$ is the Thomas-Fermi radius, and ${\tilde{E}}_{F}=\sqrt{N}\hslash {\omega }_{\perp }$ is the Fermi energy in the trapped systems. As shown in Fig. 5(a), for ${\omega }_{0}\ge {\tilde{E}}_{F}$ (fully polarized fermions), the density profile does not depend on the effective fermion-photon coupling strength *η*, which implies that the critical line $\eta ={\eta }_{c}^{\mathrm{(1)}}$ for the second-order quantum phase transition from the N-I phase to the SR phase is unaffected by the trapping potential. However, for ${\omega }_{0} < {\tilde{E}}_{F}$ (partially polarized fermions), the density profile depends strongly on the effective fermion-photon coupling strength *η*, leading to the modification of the critical line for the first-order quantum phase transition from the N-II phase to the SR phase, as shown in Fig. 5(b). Moreover, the predicted N-II-SR mixed phase only exists near the center of the trap and the corresponding density profile has thus a jump discontinuity, which provides an experimentally observable signature. We also note that the presence of the harmonic trap renders the system to become of finite size, i.e., the density profile vanishes for size $r/{R}_{T} > {r}_{c}/{R}_{T}$, where *r*_*c*_ denotes the size of the trap, as shown in both Fig. 5(a) and 5(b).

In addition, when the decay rate *κ*, which has the order of MHz in experiments, is much larger than the external atomic degrees of freedom, the relatively slow atoms at times $t\gg 1/\kappa$ feel an average affect of steady-state photons, i.e., Eq. (14). Furthermore, when the system enters the SR phase with a large mean-photon number, the commutator $[\hat{a},{\hat{a}}^{\dagger }]=1$ can be ignored at the leading order results. Thus, it is reasonable to neglect the non-commutability between $\hat{a}$ and ${\hat{a}}^{\dagger }$ and just set them to their steady-state averaged value^3, 4, 12–25^. Based on the above two arguments, the employed mean-field approximation, which assumes to approximate the photon annihilation operator by its steady-state averaged value, provides a good description of the system within our context.

In summary, we have analytically investigated the ground-state properties of a 2D polarized degenerate Fermi gas coupled to a high-finesse optical cavity. By solving the photon-number dependent BdG equation, we have found rich quantum phases and phase diagrams, which depend crucially on the fermion-photon coupling strength and the atomic resonant frequency (effective Zeeman field). In particular, for the weak atomic resonant frequency, we have shown that there exists a mixed phase where the N-II and SR phases coexist. In addition, we have revealed a first-order quantum phase transition from the N-II phase to the SR phase, which contrasts to the familiar second-order transition for the case with bosons. Finally, we have presented a parameter estimation and have addressed briefly how to detect these predicted quantum phases and phase diagrams in experiments.

## Methods

In this Section, we present a detailed derivation of Eqs (45)–(48). We denote by ${\mu }_{N-II-SR}\in [{\mu }_{N-II},{\mu }_{SR}]$ the chemical potential in the N-II-SR mixed phase, and let ${n}_{N-II}$ (*n*_*SR*_) and ${\mu }_{N-II}$ (*μ*_*SR*_) denote the atom density and the chemical potential in the N-II (SR) phase, respectively. In terms of the fraction $0\le {x}_{0}\le 1$ of the N-II phase, we have^42^

50 $$n={x}_{0}{n}_{N-II}({\mu }_{N-II-SR},{\omega }_{0})+(1-{x}_{0}){n}_{SR}({\mu }_{N-II-SR},{\omega }_{0},\eta )\mathrm{.}$$

When *x*_0_ = 1, we simply have the N-II phase [see Eq. (28)], and ${n}_{N-II}=M{\mu }_{N-II}/\pi$. Instead, when *x*_0_ = 0, ${\bar{E}}_{{\rm{G}}}^{{\rm{SR}}}({\mu }_{SR},{\omega }_{0},\eta )$, *μ*_*SR*_, ${|\bar{\alpha }|}_{SR}$, *n*_*SR*_, and ${\bar{m}}_{SR}$ are given by

51 $${\bar{E}}_{{\rm{G}}}^{{\rm{SR}}}=-\frac{{\mu }_{SR}^{2}}{4{E}_{F}A}-\frac{{\omega }_{0}^{2}({\omega }^{2}+{\kappa }^{2})}{4\omega {\eta }^{2}},$$

52 $${\mu }_{SR}=2({E}_{F}-\frac{\omega {\eta }^{2}}{{\omega }^{2}+{\kappa }^{2}}),$$

53 $${|\bar{\alpha }|}_{SR}=\frac{1}{2}\sqrt{\frac{{\mu }_{SR}^{2}{\eta }^{2}}{({\omega }^{2}+{\kappa }^{2}){E}_{F}^{2}{A}^{2}}-\frac{{\omega }_{0}^{2}({\omega }^{2}+{\kappa }^{2})}{{\omega }^{2}{\eta }^{2}}},$$

54 $${n}_{SR}=\frac{M{\mu }_{SR}}{2\pi A},$$

55 $${\bar{m}}_{SR}=\frac{{\omega }_{0}({\omega }^{2}+{\kappa }^{2})}{4\omega {\eta }^{2}},$$

where $$A=1-\omega {\eta }^{2}/[{E}_{F}({\omega }^{2}+{\kappa }^{2})]$$.

Using the phase equilibrium condition ${\bar{E}}_{{\rm{G}}}^{{\rm{N}}-{\rm{II}}}({\mu }_{N-II-SR},{\omega }_{0})=$ ${\bar{E}}_{{\rm{G}}}^{{\rm{SR}}}({\mu }_{N-II-SR},{\omega }_{0},\eta )$ ^37, 42^, together with Eqs (28) and (51), we see that when ${\mu }_{N-II-SR}={\mu }_{N-II}={E}_{F}$ corresponding to *x*_0_ = 1, the phase boundary between the N-II phase and the N-II-SR mixed phase is described by $\eta ={\eta }_{c}^{(2)}$. On the other hand, when ${\mu }_{N-II-SR}={\mu }_{SR}=2{E}_{F}-2\omega {\eta }^{2}/({\omega }^{2}+{\kappa }^{2})$ corresponding to *x*_0_ = 0, the phase boundary between the N-II-SR mixed phase and the SR phase is also described by $\eta ={\eta }_{c}^{(2)}$. In addition, for the N-II-SR mixed phase, we can determine ${\mu }_{N-II-SR}$ from the relation ${\bar{E}}_{{\rm{G}}}^{{\rm{N}}-{\rm{II}}}({\mu }_{N-II-SR},{\omega }_{0})=$ ${\bar{E}}_{{\rm{G}}}^{{\rm{SR}}}({\mu }_{N-II-SR},{\omega }_{0},\eta )$, which gives

56 $${\mu }_{N-II-SR}={E}_{F}\mathrm{.}$$

This is the same as Eq. (46). Substituting Eq. (56), ${n}_{N-II}$, and *n*_*SR*_ into Eq. (50), we find *x*_0_ can take arbitrary values ranging from 0 to 1.

Finally, let us prove that the ground state of the N-II-SR mixed phase is stable. Due to the existence of both N-II and SR phases, the scaled ground-state energy in this mixed phase is defined by^37, 42^

57 $$\begin{array}{c}{\bar{E}}_{{\rm{G}}}^{{\rm{N}}-{\rm{II}}-{\rm{SR}}}({\omega }_{0},\eta )={\mu }_{N-II-SR}+{x}_{0}{\bar{E}}_{{\rm{G}}}^{{\rm{N}}-{\rm{II}}}({\mu }_{N-II-SR},{\omega }_{0})\\ \quad \quad \quad \quad \quad \quad \quad +(1-{x}_{0}){\bar{E}}_{{\rm{G}}}^{{\rm{SR}}}({\mu }_{N-II-SR},{\omega }_{0},\eta )\mathrm{.}\end{array}$$

Thus we obtain

58 $${\bar{E}}_{{\rm{G}}}^{{\rm{N}}-{\rm{II}}-{\rm{SR}}}({\omega }_{0},\eta )-{\bar{E}}_{{\rm{G}}}^{{\rm{N}}-{\rm{II}}}({\omega }_{0})=\mathrm{0,}$$

59 $${\bar{E}}_{{\rm{G}}}^{{\rm{N}}-{\rm{II}}-{\rm{SR}}}({\omega }_{0},\eta )-{\bar{E}}_{{\rm{G}}}^{{\rm{SR}}}({\omega }_{0})=-\frac{{E}_{F}^{2}-{\omega }_{0}^{2}}{2{E}_{F}},$$

which are either less than or equal to zero, i.e., the ground state of the N-II-SR mixed phase is stable at $\eta ={\eta }_{c}^{(2)}$.

Substituting Eqs (28), (51), and (56) into Eqs (53) and (57), we derive Eqs (45)–(47). In addition, according to Eq. (50), we obtain Eq. (48).
